# Aquaporin-1 facilitates proliferation and invasion of gastric cancer cells via GRB7-mediated ERK and Ras activation

**DOI:** 10.1080/19768354.2020.1833985

**Published:** 2020-10-21

**Authors:** Zhenjie Wang, Yujuan Wang, Yuan He, Ning Zhang, Wei Chang, Yahui Niu

**Affiliations:** a Department of Digesting Internal Medicine, Changzhi City, People’s Republic of China; b Department of Ultrasound Diagnosis, Affiliated Heping Hospital, Changzhi Medical College, Changzhi City, People’s Republic of China; c Department of Gastrointestinal Surgery, Affiliated Heping Hospital, Changzhi Medical College, Changzhi City, People’s Republic of China; d Department of Ear-Nose-Throat, Affiliated Heping Hospital, Changzhi Medical College, Changzhi City, People’s Republic of China

**Keywords:** AQP-1, GRB7, RAS, ERK, gastric cancer

## Abstract

Gastric cancer, one of the most common malignant tumors of the digestive tract, is devoid of effective treatment owing to its highly invasive ability. Aquaporins (AQPs), transmembrane water channel proteins, has been shown to be involved in the malignancy of gastric cancer. This study aims to investigate the pathophysiological roles of AQP-1 in gastric cancer. We first demonstrated quantitative real-time polymerase chain reaction analysis and found up-regulation of AQP-1 in gastric cancer cell lines. Additionally, silence of AQP-1 inhibited cell proliferation via decrease of proliferating cell nuclear antigen (PCNA) and minichromosome maintenance complex component 2 (MCM2). Moreover, migration and invasion of gastric cancer cells were also suppressed by the interference of AQP-1. However, the tumorigenic mechanism of AQP-1 on gastric cancer is yet to be found. We demonstrated western blot analysis and found that knockdown of AQP-1 decreased protein expression of phospho (p)-GRB7 (growth factor receptor-bound protein 7) and led to a remarkable reduction of p-extracellular signal-regulated kinase (ERK) via inactivation of RAS. In general, our findings indicated that AQP-1 facilitates proliferation and invasion of gastric cancer cells via GRB7-mediated ERK and Ras activation, illuminating a novel AQP-1-RAS/ERK molecular axis as regulator in gastric cancer progression and suggesting potential implications in the treatment of gastric cancer.

## Introduction

Gastric cancer is one of the most common malignancies of the digestive tract, and remains high incidence and mortality in Asian, especially in China (Bar-Zeev et al. 2018). Radical surgery and chemotherapy are still the standard treatments for gastric cancer, in spite of recent development of new therapeutic strategies (Chan et al. 2016). However, serious adverse reactions of radical surgery and chemotherapy drugs limit the efficacy in gastric cancer (Galluzzi et al. 2014). The pathogenesis of gastric cancer is extremely complicated, so it is of great significance to clarify the pathogenesis of gastric cancer to find new therapeutic means and therapeutic targets.

Widely found in human tissues, aquaporins (AQPs) can transport water molecules efficiently and selectively (Verkman 2005). It is an important material basis for rapid water transport across cell membranes and plays an important role in cell metabolism, proliferation and the realization of physiological functions of various organs (Verkman 2008). A recent study has shown the close relation between AQPs with many clinical diseases (Verkman 2012). Moreover, AQP-1, member of AQPs, is involved in cell migration (Papadopoulos et al. 2008), angiogenesis (Zou et al. 2013) and tumor growth (Nico and Ribatti 2011). AQP-1 is highly expressed in tumor cells of different origins, especially in aggressive tumors (Verkman et al. 2008). AQP-1 could promote the growth and metastasis of brain glioma (Hayashi et al. 2007). The migration ability of melanoma cells with high expression of AQP-1 was significantly enhanced compared with cells with knockout of AQP-1, as well as local tumor infiltration and metastasis (Simone et al. 2018). Although AQP-1 was reported to be expressed in gastric cancer cells (Nagaraju et al. 2016), whether AQP-1 demonstrates a cancer-promoting role in gastric cancer remains to be investigated.

RAS proteins, belonging to small protein GTPase family, are major regulator of cellular events, such as cell growth, proliferation, differentiation, adhesion and migration in humans (Brossier et al. 2015). The study has shown that aberrantly activated RAS mutations, including H-RAS (Harvey rat sarcoma viral oncogene homolog), N-RAS (neuroblastoma RAS viral oncogene homolog) and K-RAS (Kirsten rat sarcoma viral oncogene homolog), were closely associated with carcinogenesis in various tumors (LeBleu et al. 2013). Oncogenic RAS mediated downstream eﬀector molecules, RAF-MEK-ERK signaling cascade, to regulate tumor cell proliferation and migration (Fey et al. 2016). Inactivation of RAS-RAF-MEK-ERK could suppress cell proliferation and migration of gastric cancer (Lin et al. 2014). The role of AQP-1 in RAS associated carcinogenesis of gastric cancer and the underlying mechanisms are poorly understood.

Here, the expression levels of AQP-1 in gastric cancer cells were firstly detected, and hereafter, the impact of AQP-1 on tumor progression of gastric cancer was then determined. The meaning results might provide new evidence for the development of novel treatment for gastric cancer.

## Materials and methods

### Cell culture

Human gastric cancer cell lines, including HGC-27, MKN74, MKN45, AGS, and human gastric epithelium cell line (GES-1), were cultured in RMPI-1640 medium supplemented with 10% fetal bovine serum (Lonza, Basel, Switzerland) at 37 ˚C with 5% CO_2_ atmosphere.

### Cell transfection

For the knockdown of AQP-1, siRNAs target AQP-1 (1#: 5′-CCACGACCCTCTTTGTCTT-3′ and 2#: 5′-GGAGGAGTATGACCTGGAT-3′) and the negative control (siNC; 5′-TTCTCCGAACGTGTCACGT-3′) were synthesized by GenePharma (Suzhou, China). AGS and MKN45 cells were transfected with 100 pM siRNAs via Lipofectamine 2000 (Invitrogen, Carlsbad, CA, USA).

### CCK8

AGS and MKN45 cells (1 × 103/well) were seeded, and then treated with 10 μL CCK-8 solution (Dojindo, Tokyo, Japan) every 24 h intervals (24, 48, 72 h) for 2 h. Microplate reader (Molecular Devices Sunnyvale, CA, USA) was used to measure the optical densities at 450 nm.

### Wound healing

AGS and MKN45 cells (1 × 10^6^/well) were seeded with a wound gaps generated by a plastic pipette tip. After removing debris or the detached cells, cells were cultured in RMPI-1640 medium for another 24 h before calculating the wound width under an inverted microscope.

### Transwell assay

AGS and MKN45 cells (5 × 10^4^/well) were seeded into the upper wells of chamber (BD Biosciences, Bedford, MA, USA) with the Matrigel-coated membrane (BD Biosciences). Migration-inducing medium (with 10% FBS) was added to the lower wells of chambers. 24 h later, the invasive cells to the lower wells of chambers were fixed with 100% methanol for 30 min, and then stained with 0.1% crystal violet for 1 h. The stained cells were imaged and counted under a microscope (Olympus).

### qRT-PCR

Total RNAs from tissues or cells were isolated with Trizol (Invitrogen), and then the RNAs were reverse-transcribed into cDNAs by PrimeScript RT Reagent (Takara, Shiga, Japan). qRT-PCR was performed via SYBR Green Master (Roche, Mannheim, Germany). GAPDH was used as endogenous control. The primer sequences were shown in Table 1.

**Table 1.** Primer.

ID	Sequence(5′–3′)
GAPDH F	ACCACAGTCCATGCCATCAC
GAPDH R	TCCACCACCCTGTTGCTGTA
AQP-1 F	TGCCATCGGCCTCTCTGTAG
AQP-1 R	AAGGACCGAGCAGGGTTAATC

### Pulldown assay

GST (glutathione S-transferase)-fused Ras-binding domain of Raf protein (GST-Raf-RBD) was precoupled with glutathione-Sepharose 4B beads (GE Healthcare, Marlborough, MA, USA) and then incubated with AGS or MKN45 cell lysates for 90 min at 4°C. After washing with phosphate-buffered saline, the beads were analyzed by western blot to detect active RAS via antibodies (Abcam, Cambridge, MA, USA). The total RAS in lysates was also evaluated by western blot.

### Western blot

Proteins extracted from cells (30 µg) were separated by SDS-PAGE, and then electro-transferred onto PVDF membrane. After blocking with 5% BSA, the membrane was incubated overnight with primary antibody: anti-PCNA, anti-MCM2 antibodies (1:1500, Abcam), GRB7 and p-GRB7 (1:2000, Abcam), ERK and p-ERK (1:2500, Abcam), GAPDH (1:3000, Abcam) at 4°C. Following incubation with horseradish peroxidase-labeled secondary antibody (1:10,000; Abcam), the immunoreactivities were detected by enhanced chemiluminescence (KeyGen, Nanjing, China).

### Statistical analysis

All results are expressed as mean ± SEM. The statistical analyses were determined via GraphPad Prism software and one-way analysis of variance. *P* < .05, *P* < .01 or *P* < .001 was considered as a mark of statistically significant.

## Results

### Up-regulation of AQP-1 in gastric cancer cell lines

**Figure 1.**
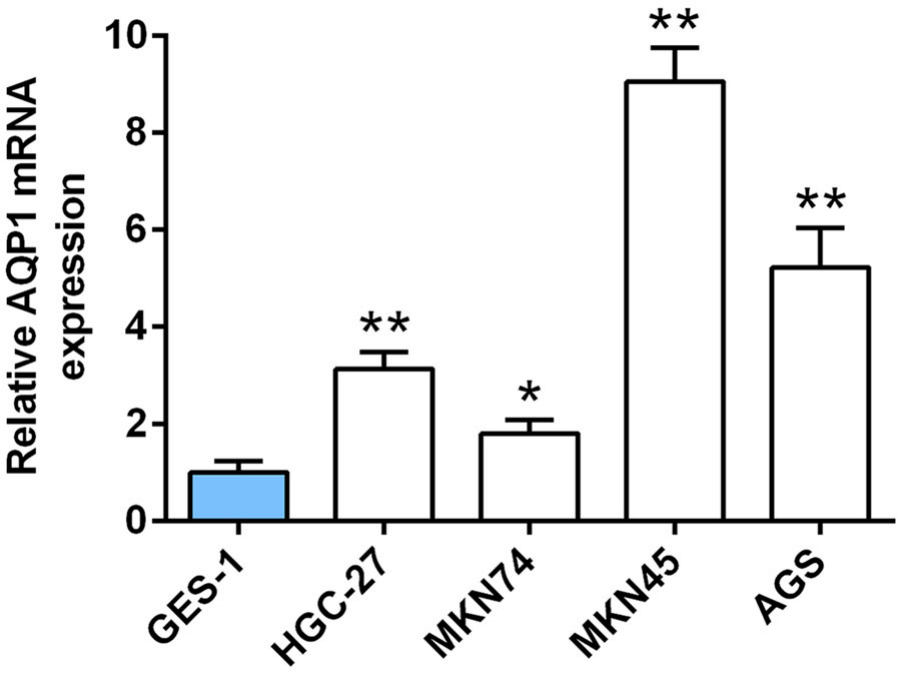
Up-regulation of AQP-1 in gastric cancer cell lines. The expression of AQP-1 in gastric cancer cell lines and GES-1 cells detected by qRT-PCR. *, ** represents gastric cancer cell lines vs. GES-1, *P*  < .05, *P* < .01.

A significantly up-regulation of AQP-1 in gastric cancer cell lines, including HGC-27, MKN74, MKN45, AGS, compared to human gastric epithelium cell line (GES-1) was verified by qRT-PCR (Figure 1), suggesting a potential correlation between AQP-1 and gastric cancer progression. AGS and MKN45 cells with the higher expression of AQP-1 were selected for the subsequent experiments.

### Interference of AQP-1 inhibited gastric cancer progression

**Figure 2.**
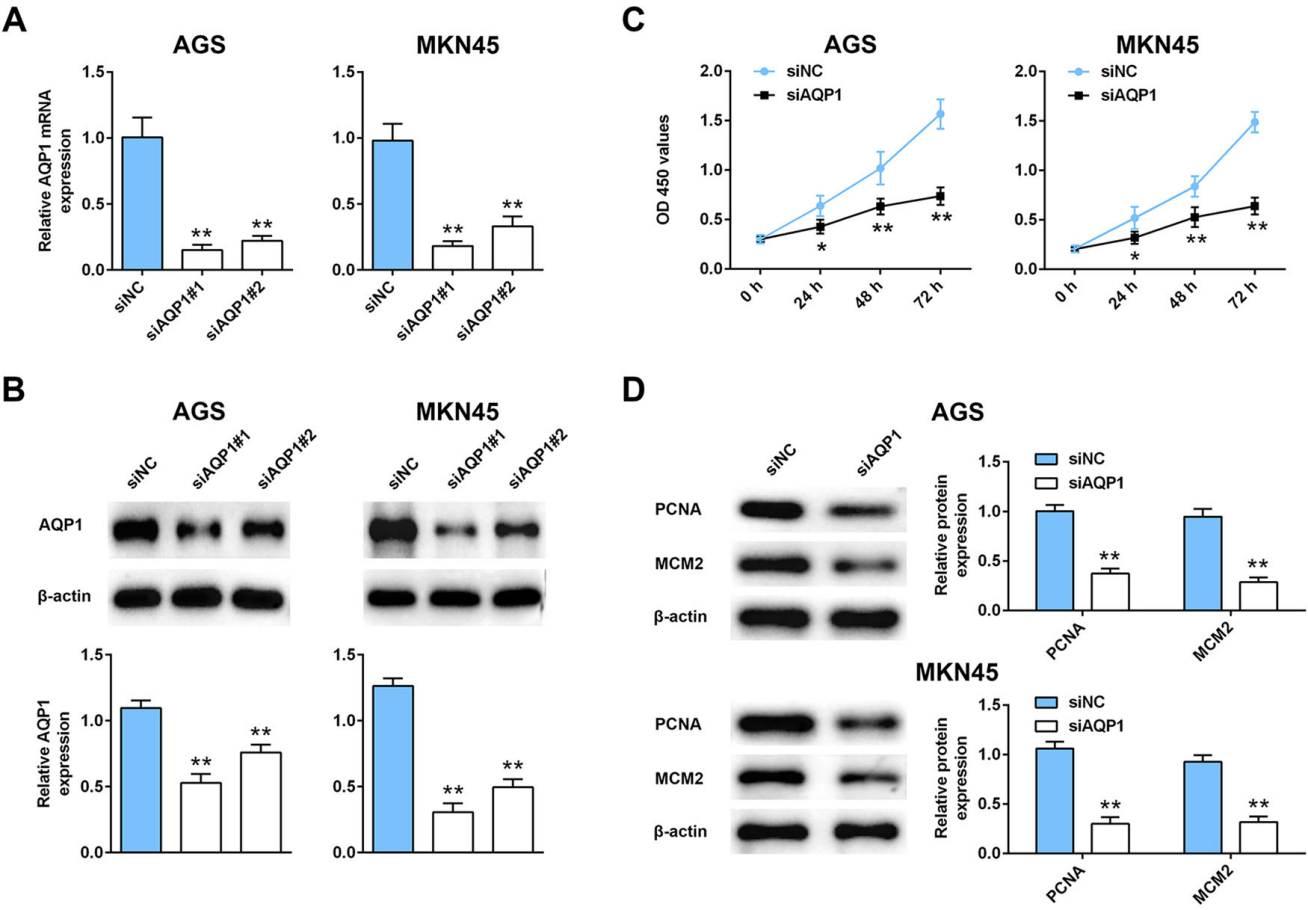
Interference of AQP-1 inhibited gastric cancer proliferation. (A) Transfection efficiency of siAQP1 #1 or #2 in AGS and MKN45 cells detected by qRT-PCR. ** represents siAQP1 #1 or #2 vs. siNC, *P* < .01. (B) Transfection efficiency of siAQP1 #1 or #2 in AGS and MKN45 cells detected by western blot. ** represents siAQP1 #1 or #2 vs. siNC, *P* < .01. (C) The effect of AQP-1 on cell proliferation of AGS and MKN45 cells detected by CCK8. *, ** represents siAQP1 vs. siNC, *P* < .05, *P* < .01. (D) The effect of AQP-1 on protein expression of PCNA and MCM2 in AGS and MKN45 cells detected by western blot. ** represents siAQP1 vs. siNC, *P* <  .01.

**Figure 3.**
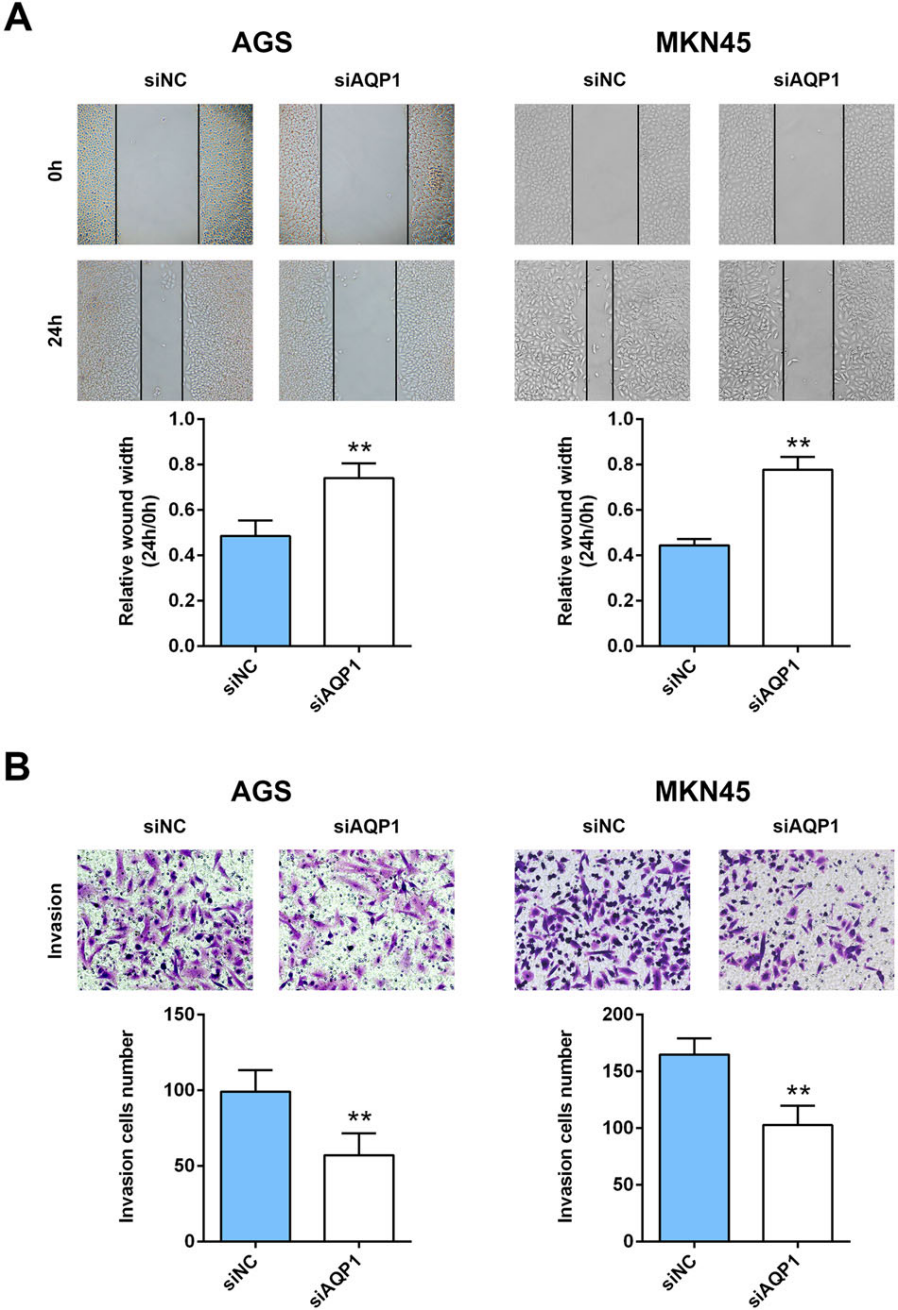
Interference of AQP-1 inhibited gastric cancer migration and invasion. (A) The effect of AQP-1 on cell migration of AGS and MKN45 cells detected by wound healing assay. ** represents siAQP1 vs. siNC, *P* < .01. (B) The effect of AQP-1 on cell invasion of AGS and MKN45 cells detected by transwell assay. ** represents siAQP1 vs. siNC, *P* <  .01.

To explore the potential effect of AQP-1 on gastric cancer progression, CCK8 and western blot assays were employed to estimate cell proliferation. Firstly, AGS and MKN45 cells were transfected with siRNAs targeting AQP-1 (siAQP1 #1 and siAQP1 #2). Both of qRT-PCR (Figure 2(A)) and western blot (Figure 2(B)) analysis indicated the silence efficiency of siAQP1 #1 and siAQP1 #2 compared to siNC. siAQP1 #1 with lower expression of AQP-1 was selected for the subsequent experiments, and named as siAQP1. Moreover, lower cell viability was discovered in AGS and MKN45 cells transfected with siAQP1 compared cells transfected with siNC (Figure 2(C)), suggesting that interference of AQP-1 inhibited cell proliferation of gastric cancer. Proteins involved in cell proliferation, including PCNA and MCM2, were down-regulated in cells transfected with siAQP1 (Figure 2(D)). Cell migration (Figure 3(A)) and invasion (Figure 3(B)) were also suppressed by the interference of AQP-1 in AGS and MKN45 cells, indicating that AQP-1 may account for the malignant phenotypes of gastric cancer.

### Interference of AQP-1 inhibited GRB7-mediated RAS/ERK activation

**Figure 4.**
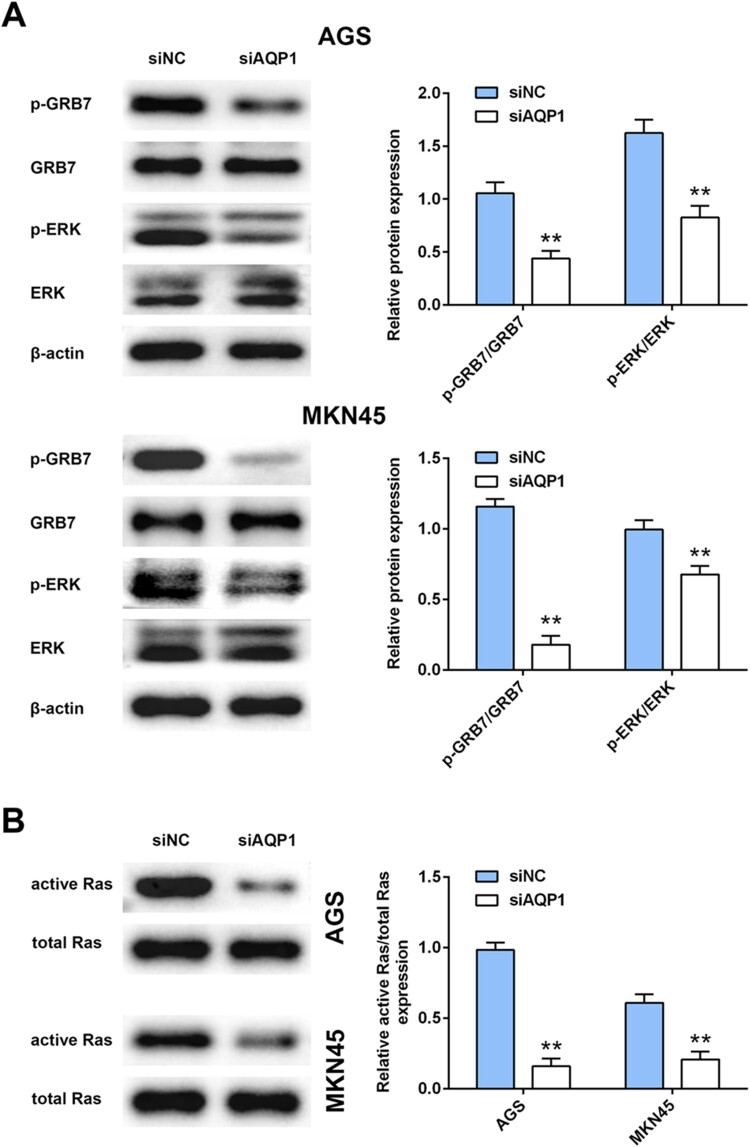
Interference of AQP-1 inhibited GRB7-mediated RAS/ERK activation. (A) The effect of AQP-1 on protein expression of GRB7, p-GRB7, ERK and p-ERK in AGS and MKN45 cells detected by western blot. ** represents siAQP1 vs. siNC, *P* < .01. (B) The effect of AQP-1 on protein expression of active and total RAS in AGS and MKN45 cells detected by western blot. ** represents siAQP1 vs. siNC, *P* < .01.

To explore the molecular mechanism of AQP-1 on gastric cancer progression, western blot analysis was then employed. Although interference of AQP-1 had no significant change on protein expression of GRB7 and ERK in AGS and MKN45 cells, AQP-1 silence decreased the activation of GRB7-mediated ERK activation, as demonstrated by decrease of p-GRB7 and p-ERK (Figure 4(A)). Moreover, total RAS protein was not affected by siAQP1, while GST pulldown showed that the active form of RAS was dramatically down-regulated in AGS and MKN45 cells transfected with siAQP1 (Figure 4(B)). These results suggested that AQP-1 was involved in RAS/ERK associated carcinogenesis of gastric cancer.

## Discussion

Ion channels, which are localized in the plasma membrane, respond to extracellular signals to participate in cell signaling and cancer progression, especially in gastric cancer (Anderson et al. 2019). Inhibitors of ion channels show anti-proliferative effect against tumors (Cai et al. 2009; Jang et al. 2011). AQPs, which are water and glycerol carriers, have also to shown be crucial for malignancy of gastric cancer (Xia et al. 2017). AQP-3 (Huang et al. 2010) and AQP-5 (Huang et al. 2013) promote migration and proliferation of gastric adenocarcinoma cells. Inhibition of AQP-3 could reduce colon cancer (Dorward et al. 2016) or pancreatic cancer (Liu et al. 2012) cell migration and invasion. Since AQP-1 could promote the rapid flow of water into the lamellar pseudopodia at the leading edge of migrating cells, thus promoting the change of cell morphology and the forward movement of tumor cells (Saadoun et al. 2005). AQP-1 may be a potential avenue for pharmaceutical research in gastric cancer.

Firstly, up-regulation of AQP-1 in gastric cancer cell lines indicated a potential role of AQP-1 in gastric cancer. *In vitro* loss- of function assays revealed that interference of AQP-1-suppressed gastric cancer proliferation, migration and invasion. Moreover, AQP-1 could regulate cell apoptosis of osteosarcoma cells (Wu et al. 2015). The functional involvement of AQP-1 in cell apoptosis of gastric cancer needs to be further studied. PCNA, critical regulator in DNA synthesis, whose expression was closely related to poor prognosis of gastric cancer patients (Lee et al. 2003). Up-regulation of PCNA promotes tumor growth in gastric cancer (He et al. 2019). Moreover, MCM2, responsible for DNA synthesis during cell cycle, predicts poor prognosis in gastric cancer (Tokuyasu et al. 2008). Inhibition of MCM2 could suppress the growth of gastric cancer (Li et al. 2013). Results in the present study showed that knockdown of AQP-1 decreased protein expression of PCNA and MCM2 in gastric cancer cells, suggesting the anti-proliferative role of AQP-1 silence in gastric cancer. Furthermore, aberrant epithelial–mesenchymal transition promotes cell initiation, invasion, metastasis in gastric cancer (Huang et al. 2015). The promotive role of AQP-3 on tumor growth of gastric cancer depends on epithelial–mesenchymal transition (Chen et al. 2014). The influence of AQP-1 on epithelial–mesenchymal transition and stemness properties of gastric cancer is also needed for further investigation.

In addition to the close involvement of AQP-1 in cancer progression, the downstream signaling pathways were then implicated in gastric cancer. Interaction of AQP1 with β-catenin is involved in the migration of mesenchymal stem cells (Meng et al. 2014). Down-regulation of Rho GTPases following by AQP-1 silence-suppressed tumorigenesis of osteosarcoma cells (Wu et al. 2015). This study found that knockdown of AQP-1 inhibited the active of another GTPase, RAS, as well as the phosphorylation of ERK. RAS/ERK signaling pathway is associated with gastric cancer (Gonzalez-Hormazabal et al. 2018). Inactivation of ERK was involved in AQP-3 silence-suppressed gastric carcinoma cells growth (Wang et al. 2012). Therefore, AQP-1 silence could inhibit gastric cancer progression through inactivation of RAS/ERK pathway. Moreover, in addition to ERK, many downstream effector signaling pathways, such as phosphatidylinositol 3-kinase-AKT pathway, phospholipase C- protein kinase C pathway, are involved in the regulation of RAS on tumor progression (Khan et al. 2019). Therefore, other downstream effector signaling pathways of RAS should also be investigated in AQP-1-mediated gastric cancer. Furthermore, AQP1 exhibits its tumorigenic role through activation of FAK signaling (Tomita et al. 2017), and FAK could phosphorylate GRB7 to promote tumorigenesis (Chu et al. 2009). GRB7 could bind with RAS and promote its activation for the tumorigenicity of breast cancer (Chu et al. 2010). Results in this study showed that phosphorylation of GRB7 were inhibited by knockdown of AQP-1, suggesting that Aquaporin-1 facilitates proliferation and invasion of gastric cancer cells via GRB7-mediated ERK and Ras activation.

In conclusion, we showed that AQP-1 demonstrated oncogenic effects on gastric cancer via GRB7-mediated Ras/ERK activation. The present study suggested that AQP-1 might serve as a new potential therapeutic option in gastric cancer treatment through gene silence of AQP-1, AQP-1 target inhibitors or monoclonal AQP-1 specific antibody. However, promoter of AQP1 gene contains E-box/ChoRE transcriptional element (Hayashi et al. 2007), and HIF-1α could contribute to transcriptional activation of AQP-1 during hypoxia-induced AQP1 expression (Abreu-Rodríguez et al. 2011) via E-box/ChoRE element (Tomita et al. 2017). Since hypoxia-induced AQP-1 was reported to promote prostate cancer progression (Tie et al. 2012), the possible mechanism of AQP-1 upregulation in gastric cancer cells might also through E-box/ChoRE element.

## Data Availability

All data generated or analyzed during this study are included in this published article.
